# Simple Yeast-Direct Catalytic Fuel Cell Bio-Device: Analytical Results and Energetic Properties

**DOI:** 10.3390/bios11020045

**Published:** 2021-02-11

**Authors:** Mauro Tomassetti, Emanuele Dell’Aglio, Mauro Castrucci, Maria Pia Sammartino, Luigi Campanella, Corrado Di Natale

**Affiliations:** 1 Department of Chemistry, First University of Rome “La Sapienza”, P.le A. Moro 5, 00185 Rome, Italy; emanuele.dell.aglio@gmail.com (E.D.); mauro.castrucci@libero.it (M.C.); mariapia.sammartino@uniroma1.it (M.P.S.); luigi.campanella@uniroma1.it (L.C.); 2 Department of Electronic Engineering, University of Rome “Tor Vergata”, Via Politecnico 1, 00133 Rome, Italy; dinatale@uniroma2.it

**Keywords:** direct catalytic fuel cell, coupled yeast cells, glucose sensing, power source

## Abstract

This paper reports the analytical detection and energetic properties of a glucose-fed Direct Catalytic Fuel Cell (DCFC) operated in association with yeast cells (Saccharomyces Cerevisiae). The cell was tested in a potentiostatic mode, and the operating conditions were optimized to maximize the current produced by a given concentration of glucose. Results indicate that the DCFC is characterized by a glucose detection limit of the order to 21 mmol L^−1^. The cell was used to estimate the “pool” of carbohydrate content in commercial soft drinks. Furthermore, the use of different carbohydrates, such as fructose and sucrose, has been shown to result in a good current yield.

## 1. Introduction

Fuel cells are promising devices for small-scale power generation (fuel cells as energy generator). As the produced energy depends on the amount of fuel, they also find application as chemical sensors (fuel cells as sensors).

A noticeable number of studies have been reported in the literature concerning fuel cells of different types and configurations which evaluate the conversion of chemical energy into electrical energy, using hydrogen or more complex compounds as fuel, such as methanol, ethanol, or glucose [1,2,3]. Fuel cells have also been used in the past by present authors to quantify alcohol content in foods, drinks, and pharmaceutical preparations [4,5,6], while in this paper authors investigated the analytical survey of the Direct Catalytic Fuel Cell (DCFC) fuel cell for glucose determination, not neglecting to evaluate the energy production from glucose solution.

Several types of glucose-based fuel cells have been reported in the literature, such as direct abiotic fuel cells which use gold-platinum as a catalyst [7,8,9]; these devices were also proposed as implantable abiotic glucose fuel cells [10,11]. Among fuel cells, those based on biological processes are particularly appealing for their inherent sustainability. Enzymatic glucose biofuel cells were fabricated [12,13,14], using enzymes such as glucose oxidase and laccase as electrocatalysts, however they have limited stability [8]. Lastly microbial fuel cells have been studied, where the different systems of a whole electroactive micro-organism were used [15,16,17]. Among fuel cells, those based on biological processes are particularly appealing for their inherent sustainability. In these devices, microorganisms are used as the primary engine of biological processes for energy production. In the present paper we emphasized the use of a simple commercial DCFC device, coupled with Saccharomyces Cerevisiae cells, for analytical purpose, i.e., for glucose determination. As reported in the reference [17] of the present paper, other researchers also addressed the same topic, especially to investigate energy production. However, as can easily be seen, reading the paper cited in reference [17], practically all these researchers have worked on rather complex systems, typically involving the immobilization of yeast in the anodic compartment of the fuel cell along with an exogenous mediator, like methylene blue, or bromocresol, or eriochrome, and so on. Initially, we also experimented with devices based on similar configurations but, due to the lackluster results obtained, the difficulties of their realization, and above all the short duration of these devices, we immediately opted for a simpler, more practical, and above all more durable device. We have, in fact, noticed that the immobilization of yeast in the anodic sector of the cell necessarily requires the use of a very limited quantity of yeast (and this does not represent an advantage, given the very low cost of commercial yeasts, such as Saccaromyces Cerevisiae), which involves a very low reproducibility of the measures and limits, almost entirely, the use of the device for analytical purposes. This is due to the fact that any immobilized yeast cell—statistically removed from the support—tends to clog the separation membrane, rapidly decreasing the ionic permeability and thus also limiting the life of the device from an energy point of view. On the contrary, the device described in our work involves the use of yeast placed in an external thermostated vessel, in addition to an efficient filtering system that does not allow the penetration of any yeast cells (or other particles) in the anodic compartment of the fuel cell. This makes measures much more repeatable, especially for analytical purposes, and increases the use of the same fuel cell, practically for an unlimited time. In addition, the latter can also be a simple inexpensive fuel cell available on the market (as in our case). It is clear that even in our device it is possible to immobilize the yeast, but in this case, this occurs in the external vessel. We performed tests using immobilized yeast placed in a special thermostated column, but soon gave up the immobilization. We preferred to use it free in solution under slight stirring because, using a commercial yeast, its cost was practically negligible. Furthermore, it was possible to thermostat the system as well as the possibility of easily optimizing the various operating parameters, which brought great advantages to the efficiency and repeatability of the measurement—very useful especially in analytical applications.

In the present research, we also investigated the interplay between the energy production and the glucose quantification in a Yeast Direct Catalytic Fuel Cell (YDCFC). In practice, the fuel cell exploited the fermentation reaction induced by yeast cells (Saccaromyces Cerevisiae in our case) by converting glucose into ethanol through the following reaction:

$Glucose\;\left(1\;mol\right)\overset{\left(Yeast\right)}{\to }\mathrm{Pyruvate}\;\left(2\;\mathrm{mol}\right)\;\to \;\mathrm{Acetaldehyde}\;\left(2\;\mathrm{mol}\right)\;\to \;\mathrm{Ethanol}\;\left(2\;\mathrm{mol}\right)$ The produced ethanol was then used as the actual fuel for the DCFC device resulting in electric energy. This paper shows that the system can be used both for glucose detection and to estimate the energy production.

## 2. Materials and Methods

The solutions used for all the experiments were made by dissolving in 30 mL of distilled water; 0.34 g of Glycine (Fluka, Seelze, Germany, assay > 99%). Glycine solution (30 mL) was used to make an isotonic solution (0.15 mol L^−1^, pH = 6.0) with 0.6 g of yeast (Saccharomyces Cerevisiae). Glucose D (+)-Glucose Monohydrate was purchased from Fluka (Seelze, Germany, assay > 99%), while commercial yeast preparation was purchased from a local shop.

A Mettler PM460 balance (Columbus, OH, USA) was used for weighing all solid products.

**Figure 1:**
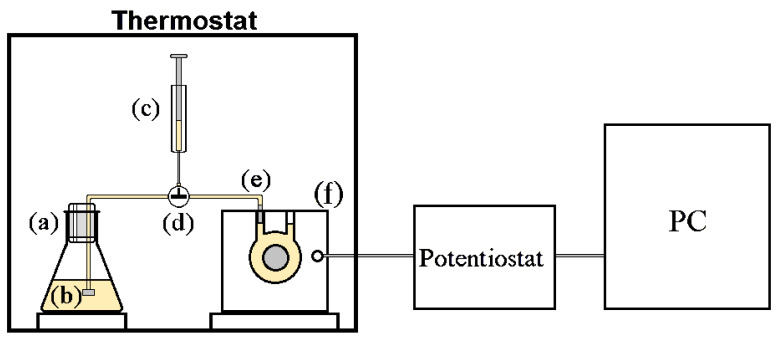
Experimental apparatus: (**a**) flask containing glucose and yeast in glycine solution; (**b**) first small filter; (**c**) graduated syringe; (**d**) turning tap; (**e**) second small filter; (**f**) catalytic fuel cell.

Figure 1 shows the experimental apparatus used for batch measurements.

A 50 mL glass flask, properly closed with a glass stopper and filled with 30 mL of yeast-glucose-glycine solution, was kept at a constant temperature of either 25 ± 0.5 °C, for 24 h, or 28.5 ± 0.5 °C, for 12 h. The yeast was kept in suspension by a magnetic stirrer, set at 100 rpm.

At the end of the incubation time, 2 mL of solution was quickly extracted from the flask with a device made by graduated syringe, equipped with two small filters (see Figure 1), and injected in the DCFC. The cell was a HTEC Model F111 fuel cell (50 × 50 × 40 mm and weighing 100 g), produced by Fuel Cell Store (College Station, TX, USA).

The Cell walls were in Plexiglas^©^ and the end plate of the electrode was made of a Pt-Ru black catalyst, assembled with a Nafion™ membrane.

The cell was used in a potentiostatic mode by measuring the so-called supplied current [4,5,18]. For this purpose, an EmStat potentiostat from Palmsens, connected to a PC, was used. The PSTrace Software (release 4.6) to acquire data and to set the potentiostat, was used. The fuel cell Pt-Ru anode was connected to the potentiostat as a working electrode, while the Pt cathode was connected to both the reference and the counter electrodes. The open circuit voltage (OCV) was measured for approximately 200 s before any measurement, after that the anode potential was set to OCV-100 mV [4,5]. The supplied current was recorded after 60 min from the injection of the solution in the fuel cell. Before any measurement, the fuel cell was rinsed, firstly with 0.5% ethanol-water solution, then by distilled water.

## 3. Results

First, the glucose sensing properties of the fuel cell were investigated. Using the apparatus illustrated in Figure 1, adding glucose alone to the solution in the flask, then injecting this solution into the fuel cell without any previous yeast treatment, no readable current signal was observed. Therefore, the electromotive force and the resulting current appear only after the addition of yeast cells (Saccharomyces Cerevisiae) to the glucose solution contained in the flask.

**Figure 2:**
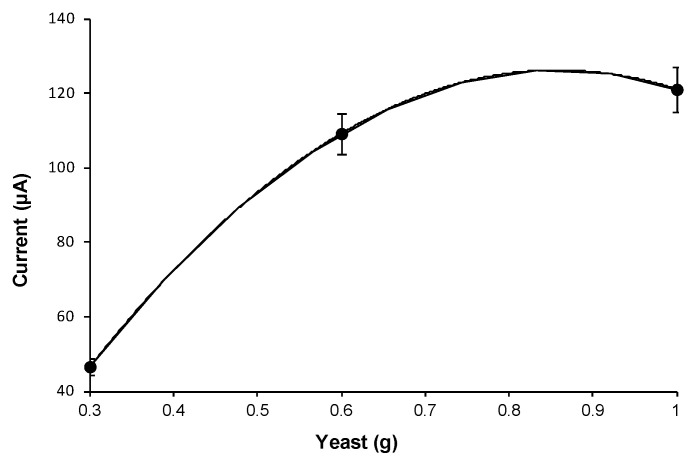
Current intensity recorded as the amount of yeast (g) in solution increases, for a fixed glucose concentration of 0.056 mol L^−1^.

**Figure 3:**
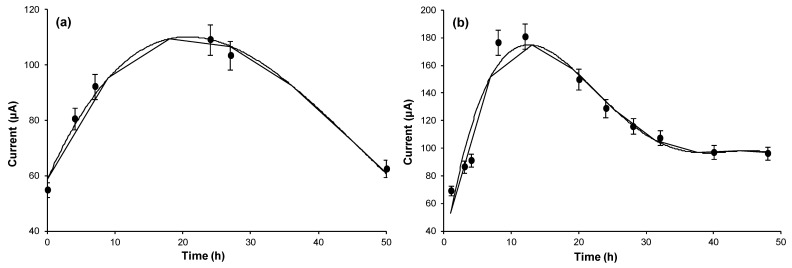
Current intensity recorded as a function of the contact time between yeast cells and glucose (**a**) operating at 25 °C; (**b**) operating at 28.5 °C. Yeast concentration: 0.6 g in 30 mL of aqueous glycine solution; glucose concentration 0.056 mol L^−1^.

**Figure 4:**
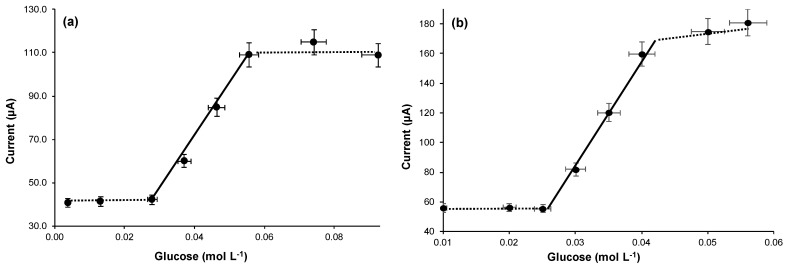
Behavior of current as a function of the increase in glucose concentration (**a**) operating at 25 °C; (**b**) operating at 28.5 °C, for a fixed yeast concentration (0.6 g in 30 mL of aqueous glycine solution).

Lastly the operating conditions were optimized by running the DCFC with the yeast cells in isotonic glycine solution at room temperature. Accordingly, the fuel cell current, at fixed and maintained constant glucose concentration, was measured as a function of the increasing quantity of yeast added (Figure 2), or of the increasing contact time (Figure 3a), by operating at 25 °C, for the best production of ethanol and to maximize the electromotive force. As can be seen in the curves reported respectively in Figure 2 and Figure 3a, it was found that 0.6 g of yeast in 30 mL glucose solution and 24 h of contact time, resulted in best conditions. Using these best conditions, the fuel cell response, with respect to the increase in glucose concentration in the aqueous glycine solution contained in the flask, was measured in order to obtain the calibration curve reported in Figure 4a. This calibration curve was obtained by measurements performed using the fuel cell operating in optimal conditions, at constant temperature of 25 °C and with the procedure described in the chapter “Material and Methods”.

It is not difficult to recognize in the curves of Figure 2, Figure 3 and Figure 4 the typical trends that correspond to the optimization of an enzymatic process. For example, the trends of the calibration curves in Figure 4 are exactly typical trends of enzyme reaction in a system in which the concentration of the enzyme is fixed and maintained constant. In this case the increase in the quantity of substrate causes an increase in the speed of the enzymatic reaction until reaching a current plateau, where the current remains constant. Furthermore, in the case of the other curves (Figure 2 and Figure 3) there is a plateau or bell-like trend, since, as in the case of enzymatic reactions, a limiting factor intervenes; for example, in the case of Figure 2, the limiting factor is the concentration of the substrate (glucose) which therefore remains constant. While similarly, in Figure 3, it is clear that, as the enzyme-substrate contact time increases, the concentration of the ethanol produced increases and consequently also the current produced by the fuel cell. At the end of the curve there is a decrease in the response, since over time some yeast cells tend to deteriorate, so even the functioning of the enzymatic system (which is the yeast’s motor) tends to slow down, decreasing the ethanol produced and therefore the current signal of the fuel cell.

Table 1 lists the linearity range and all the main analytical data of calibration curve.

Most features of the fuel cell as glucose sensors can be summarized as follows: the width of the linearity range is slightly less than 1/2 decade (between about 26 and 56 mmol L*^−^*^1^ of glucose) and the low detection limit is approximately 22 mmol L*^−^*^1^ of glucose.

It can be seen as the output current, with a glucose concentration of about 0.056 mol L*^−^*^1^, is of the order of 110 µA. This glucose concentration corresponds to the saturation of the response curve (see Figure 4a). The output current was compared with that obtained using standard ethanol concentration (see Figure 4b of work in Ref. [4] of present paper). From this value it was estimated that from a glucose solution of 0.056 mol L*^−^*^1^ about 0.004 mol L*^−^*^1^ of ethanol was produced. At this glucose concentration the generated current was about 110 µA corresponding to approximately 200 µW of electrical power.

To this point we considered some literature results, which suggested that the rate of the described reaction—catalyzed by yeast—improves, increasing the temperature [19]. 

**Figure 5:**
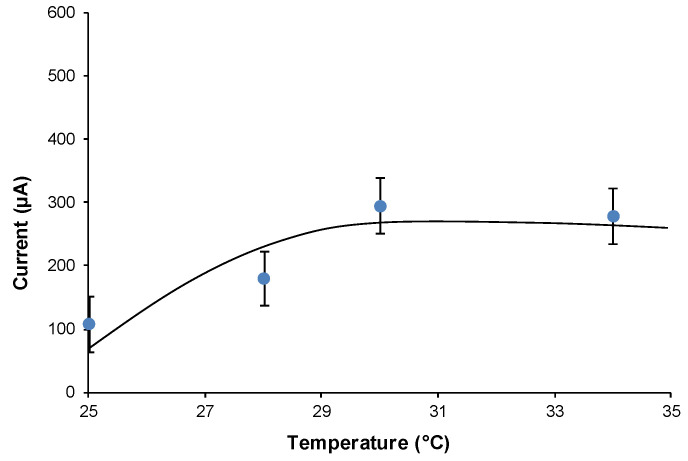
Current intensity recorded as the incubation temperature increases, obtained using 56 mmol L^−1^ of glucose concentration and 0.6 g in 30 mL of yeast.

Figure 5 shows the current, at constant glucose concentration (about 56 mmol L*^−^*^1^), vs. temperature. It shows that from 25 °C to 30 °C, the current value was about duplicate, remaining almost stable above 28.5 °C. But more interestingly, the incubation time, which needed to reach the maximum current, decreased noticeably if the incubation temperature increased from 25 °C to 28.5 °C, as can be observed in Figure 3b, which shows the current output as a function of the incubation time, recorded while keeping the cell at 28.5 °C. In fact, compared to the curve in Figure 3a, measured at 25 °C, with the curve in Figure 3b, we can see that the peak at 28 °C is reached after only 12 h of incubation time, i.e., in half time respect to the one necessary at 25 °C. While, in practice, the analytical properties of the cell did not change noticeably (see the comparison in Table 1) with the small increase of temperature (+3.5 °C), but the incubation time, to reach the maximum of the output current, was definitely shorter.

In this case too, is easy to believe that the enzymatic activity, present in the yeast, tends to increase with temperature, but then (around 28–30 °C) the activity tends to stabilize and, if we go over this temperature, the curve could go down, as the yeast would start to deteriorate.

The response of the glucose-fed cell was compared (using the optimized experimental condition) to those obtained using four different carbohydrates: xylose, fructose, galactose, and sucrose. The output current using 0.03 mol L*^−^*^1^ of each carbohydrate shows that the largest current value is obtained from sucrose disaccharide (195.69 ± 0.03 µA). While two of three other considered monosaccharides gave currents from 77 ± 0.03, to 82 ± 0.03 µA, except the case of fructose which produced about 119.06 ± 0.0 µA.

Finally, the fuel cell was used as a sensor to estimate the pool of total carbohydrates (expressed as glucose concentration) in three commercial soft drinks, tested as such, after dissolving 0.34 g of glycine in 30 mL of each beverage sample, adding 0.6 g of yeast, then incubating at 28.5 °C for 12 h, lastly recording the output current from fuel cell (μA) after 60 min from the injection of the beverage in the cell. The achieved analytical results are listed in Table 2, where the fuel cell estimates are compared with those obtained from standard solutions of glucose and ethanol respectively.

Although Table 2 does not contain the traditional results of a proper analysis, since the nominal values of carbohydrates, contained in soft beverages, were not provided by producer firms, however, it can be concluded that, our device is able to estimate carbohydrate concentration in real soft drinks, or at least to provide an estimate of the “pool” of carbohydrates present, expressing the found “pool” concentration, as mol L*^−^*^1^ glucose.

## 4. Discussion

The main results obtained can be listed as follows:

(a) From an analytical point of view, the width of the linearity range of the method to check glucose, was slightly less than 1/2 decade. The minimum detection limit was about 21 mmol L^−1^ of glucose, by operating at 28.5 °C.
(b) The present research was carried out at 25 °C, (i.e., at room temperature using a thermostat), in order to verify if the system can operate at this temperature (also without thermostat control) in a closed environment, where the change of temperature can be at most about 1 °C). In fact, as already observed in previous research [4,5], where the fuel used was only ethanol, even in the present research, where we used glucose as fuel, it was possible to perform measures at room temperature (but in a closed environment [4]) without thermostating. In these operating conditions the reproducibility did not deteriorate more than 1.5 times, compared to when the measurement was carried out in well thermostated mode.
(c) As expected, by carefully increasing the thermostating temperature of the measurement, it could be possible to shorten the measurement time, however investigation carried out in present research, which varied operating temperature conditions, has shown that, by thermostating the system at 28.5 °C (instead 25 °C), the incubation time is reduced from 24 to about 12 h, with the important benefit of shortening the measurement time.
(d) In addition, it has been experimentally verified that the system we proposed is also able to respond to several carbohydrates other than glucose. Lastly it was found suitable to estimate the carbohydrates “pool” of real samples (soft drinks) containing, in addition to glucose, also other types of carbohydrates, such as sucrose, fructose and so on.
(e) The conversion of chemical energy into electrical energy was shortly studied by comparing the current value (μA) obtained from a glucose solution at a concentration of 0.056 mol L^−1^, with the same obtained from a standard concentration of EtOH solution. It can be concluded that, from a glucose solution equal to about 0.056 mol L^−1^ and after 12 h of incubation time, about 0.004 mmol L^−1^ of EtOH can be obtained. Thus, 0.056 mol L^−1^ glucose concentration generates approximately 110 μA, i.e., it supplies a power of about 200 μW.

## 5. Conclusions

It has been experimentally shown that a yeast fuel cell is suitable for both analytical purposes (glucose determination) and energy production using glucose as fuel. It was also demonstrated that the system responds not only to glucose, but also to several other carbohydrates, such as sucrose, fructose, galactose, and xylose. These results, from an analytical point of view, encourage us to move forward in this research, by applying the method to other real samples containing glucose, or other carbohydrates, using the optimized conditions. It is also clear that this result implies that our fuel cell–yeast system can be extended to other types of substrates containing different carbohydrates. Of course, this last circumstance could also be very useful in the energy conversion field, to produce electricity by converting the chemical energy of rather cheap carbohydrates such as sucrose. The next development of the present research will concern the possibility of carrying out measurements in flow conditions rather than in batch. Some experiments in this sense have already been carried out by us and have shown that, by working under flow conditions, it certainly does not improve analytical performance, however this operating mode is very important from an energy production point of view.

## Figures and Tables

**Table 1:** Main analytical data using Direct Catalytic Fuel Cell (DCFC) as glucose sensor. Equation of calibration curves as current vs. glucose concentration.

Working Conditions: Temperature	Incubation Time	Regression of Straight Line (y = a x + b), Where: x = Glucose Concentration (mol L^−1^); y = Current (µA)	R^2^	Linearity Range (mmol L^−1^ of Glucose)	LOD (mmol L^−1^)	Pooled SD
Batch, 25.0 °C	24 h	a (slope) = 2435.9 (±120.8) b (intercept) = −27.2 (±1.3)	0.9948	26–56	22	2.9
Batch, 28.5 °C	12 h	a (slope) = 7030.4 (±351.5) b (intercept) = −123.9 (±6.1)	0.9922	25–43	21	4.5

**Table 2:** Analytical results of three analyzed drinks containing carbohydrates compared to standard solutions of glucose and ethanol.

Sample	Recorded Current (µA) (RSD% ≤ 4)	Found Concentration of Ethanol (mol L^−1^) (RSD% ≤ 5)	Found Concentration of “pool” Carbohydrates (Expressed as mol L^−1^ of Glucose) (RDS% ≤ 5)
Drink 1 (containing carbohydrates as sucrose and glucose). Incubation time 12 h	475.98	0.020	0.286
Drink 2 (containing carbohydrates as sucrose and glucose). Incubation time 12 h	323.18	0.014	0.194
Drink 3 (containing carbohydrates as sucrose and glucose). Incubation time 12 h	456.57	0.019	0.274
Pure Glucose solution (nominal value of glucose as mol L^−1^). Incubation time 12 h	169.95	-	0.056
Ethanol solution (concentration as mol L^−1^)	93.24	0.004	-

## Data Availability

Data is contained within the article.
